# Successful one-stage laparoscopic procedure for de Garengeot hernia: a totally extraperitoneal repair-first approach

**DOI:** 10.1093/jscr/rjaa586

**Published:** 2021-01-29

**Authors:** Ryota Sakon, Yoshiaki Hara, Yuki Tomizawa, Yasuhiro Ishiyama, Shingo Ito, Masataka Oneyama, Manabu Amiki, Kazuhiro Narita, Yuji Tachimori, Manabu Goto

**Affiliations:** Department of Surgery, Kawasaki Saiwai Hospital, Kawasaki-city, Kanagawa, Japan; Department of Surgery, Kawasaki Saiwai Hospital, Kawasaki-city, Kanagawa, Japan; Department of Surgery, Kawasaki Saiwai Hospital, Kawasaki-city, Kanagawa, Japan; Department of Surgery, Kawasaki Saiwai Hospital, Kawasaki-city, Kanagawa, Japan; Department of Surgery, Kawasaki Saiwai Hospital, Kawasaki-city, Kanagawa, Japan; Department of Surgery, Kawasaki Saiwai Hospital, Kawasaki-city, Kanagawa, Japan; Department of Surgery, Kawasaki Saiwai Hospital, Kawasaki-city, Kanagawa, Japan; Department of Surgery, Kawasaki Saiwai Hospital, Kawasaki-city, Kanagawa, Japan; Department of Surgery, Kawasaki Saiwai Hospital, Kawasaki-city, Kanagawa, Japan; Department of Surgery, Kawasaki Saiwai Hospital, Kawasaki-city, Kanagawa, Japan

## Abstract

The de Garengeot hernia is a femoral hernia in which the appendix migrates into the hernia sac. It is usually diagnosed intraoperatively due to its rarity and lack of clinical presentation typical to acute appendicitis. Although most cases need emergency operation due to incarceration, no standard procedure exists. We report the case of a 49-year-old woman who was diagnosed with a de Garangeot hernia preoperatively by contrast-enhanced computed tomography. She underwent one-stage laparoscopic surgery via a totally extraperitoneal approach followed by laparoscopic appendectomy. She recovered uneventfully and was discharged on postoperative Day 3. Generally, hernioplasty and appendectomy are required for the de Garengeot hernia treatment. Avoiding a peritoneal incision around the herniation and performing a mesh repair prior to appendectomy is expected to carry a lower infectious risk than other laparoscopic procedures. With accurate diagnosis, this procedure could be a useful modality for de Garengeot hernia.

## INTRODUCTION

Femoral hernias account for ~3% of all hernias. The de Garengeot hernia refers to a femoral hernia that contains the appendix, first described in 1731 by Rene Croissant de Garengeot [1]. This hernia is very rare, with an incidence of 0.5–5% of all femoral hernias. Acute appendicitis presenting within a femoral hernia is even rarer, occurring in 0.08–0.13% [2]. Although most cases require emergency operation due to incarceration, no standard procedure exists. We present the case of a 49-year-old woman who was preoperatively diagnosed with de Garengeot hernia using contrast-enhanced computed tomography (CT). She was treated by a one-stage laparoscopic surgery that was combined with a totally extraperitoneal (TEP) repair and laparoscopic appendectomy.

## CASE PRESENTATION

**Figure 1:**
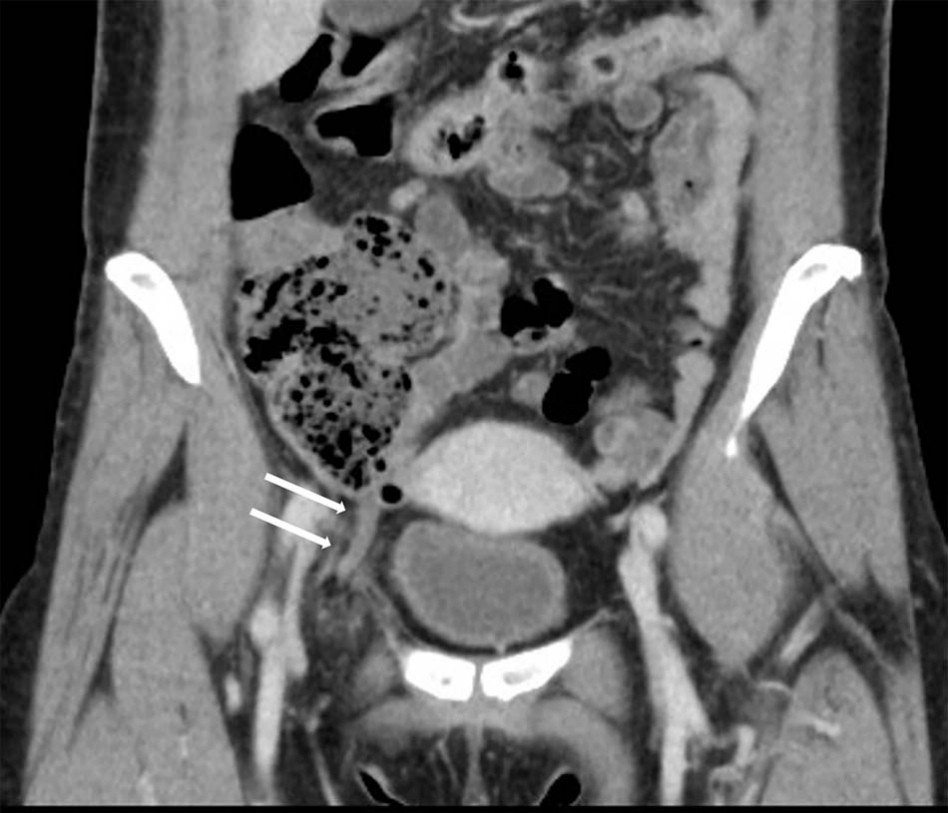
Coronal view in the abdominal CT scan shows the appendix inside the right femoral canal.

A 49-year-old woman presented to the emergency department with a 2-day history of a painful lump in the right groin. The patient did not complain fever, nausea, vomiting or a change in the bowel habits. Her medical history included pneumonia and chronic gastritis. Her general and nutritional status was good. Physical examination revealed a lump with local tenderness in the right groin below the inguinal region. There were no symptoms of dermatitis or peritonitis. Laboratory data revealed mild inflammation (white blood cells: 10 600/μl, C-reactive protein: 0.03 mg/dl). Abdominal CT revealed a blind-ended structure without an abscess inside the femoral canal. The mass was identified as a de Garengeot hernia (Fig. 1). Thereafter, an emergency surgery was scheduled; as CT did not reveal any signs of perforation, a one-stage laparoscopic procedure with a TEP-first approach was performed.

**Figure 2:**
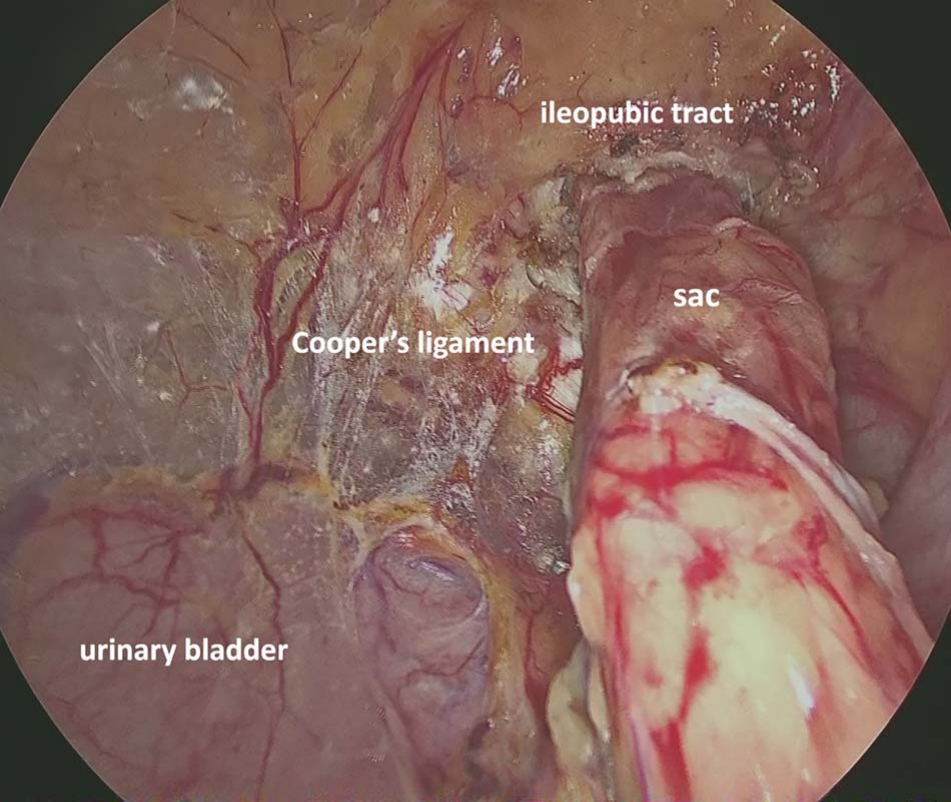
Preperitoneal view of the incarcerated hernia sac into the femoral ring.

**Figure 3:**
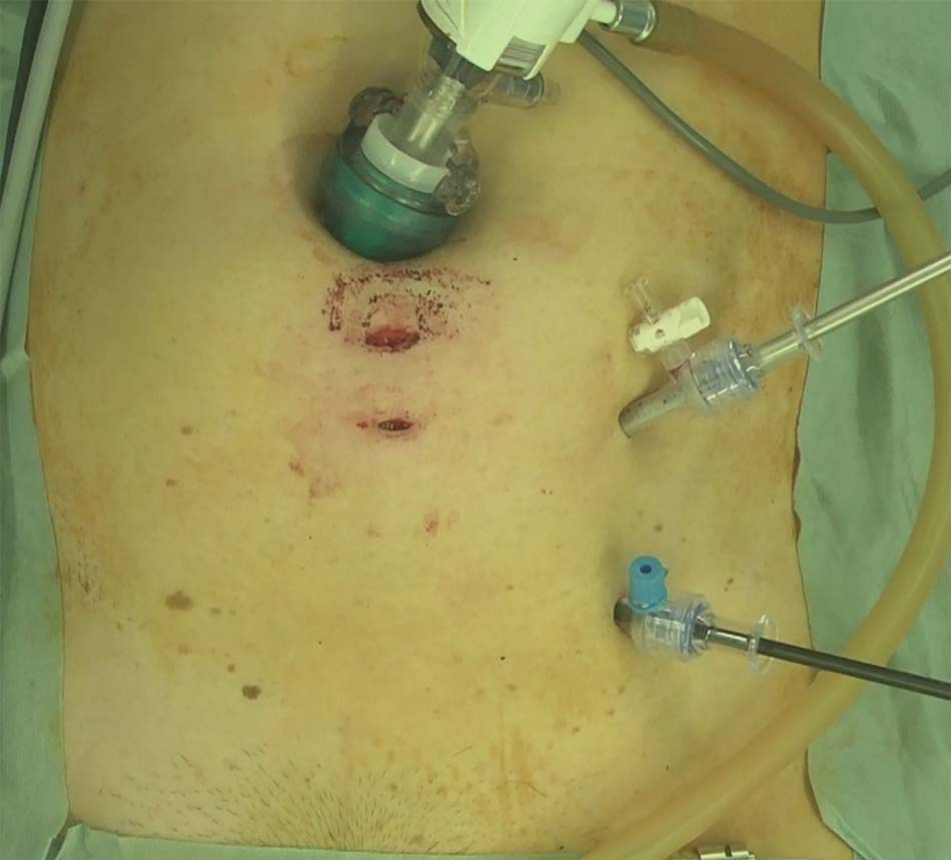
The working trocars are replaced to the left lower abdomen for the intra-abdominal operation.

**Figure 4:**
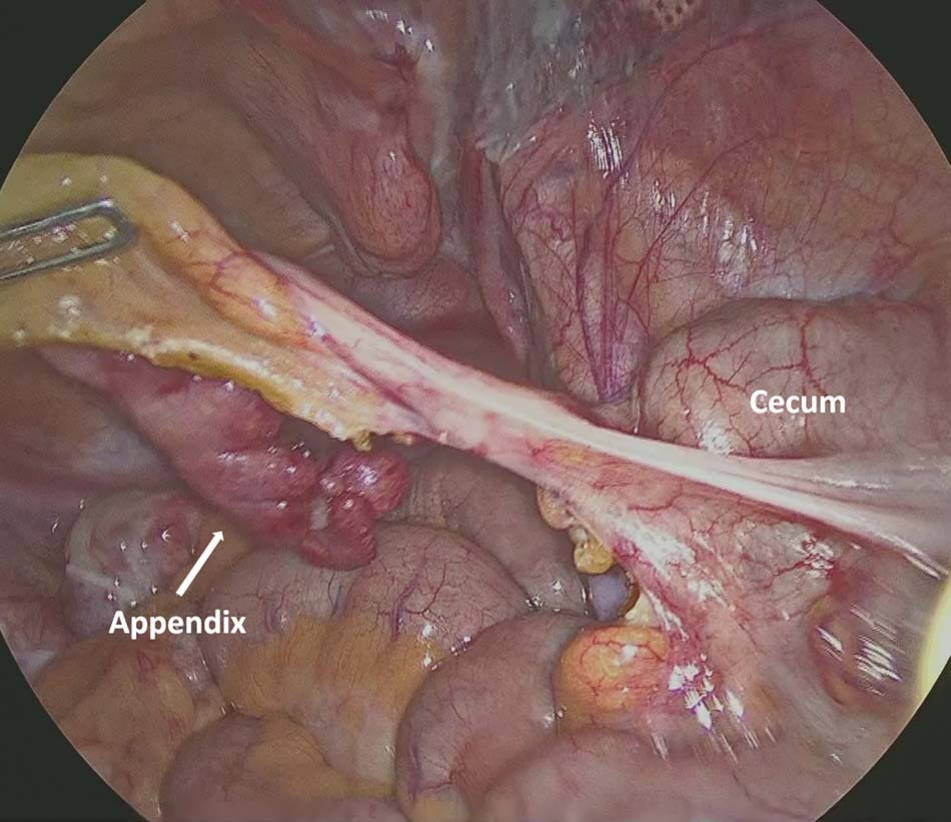
Intra-abdominal view shows the inflamed appendix without any symptoms of necrosis, perforation or abscess formation.

A 1.5-cm intraumbilical incision was made under general anesthesia, and the subcutaneous tissue and the anterior rectus sheath were dissected. The right rectus abdominis was moved to the lateral side, and a 12-mm trocar was inserted into the extraperitoneal space. Thereafter, two 5-mm working trocars were inserted into the midline of the lower abdomen. The insufflation pressure was set at 10 cm H_2_O. Under a 30-degree telescopic vision, the preperitoneal space was dissected to the inguinal region using an ultrasonically activated device. This revealed the incarcerated hernia sac in the femoral canal (Fig. 2). Because replacing the sac from the femoral canal was difficult, the lacunar ligament was divided. After the femoral ring widened, the sac was safely repositioned into the abdominal space; a 10 × 13 cm mesh (SURGIMESH XD®, Neomedica Co., Roma, Mexico) was placed without tackers to cover the space. Following the preperitoneal approach, we reinserted the 12 mm trocar into the abdominal cavity, and the other trocars were replaced to the left lower abdomen (Fig. 3). Exploration of the abdominal cavity revealed an inflamed appendix, and erythema was clearly recognized in the distal half, proving that appendicitis was caused by compression. There were no findings of necrosis, perforation or abscess formation (Fig. 4). Appendectomy was performed safely. Additionally, mesh repair was confirmed as successful through the peritoneum and there were no other coexisting hernia. The patient recovered uneventfully and was discharged on postoperative Day 3. Histopathological examination revealed moderate inflammation of the appendix.

## DISCUSSION

The de Garengeot hernia is a femoral hernia in which the appendix migrates into the hernia sac. The major risks factors of the de Garengeot hernia include the female sex (female: male; 5:1), old age, pregnancy-associated body changes, increased intra-abdominal pressure, smoking and collagen disease [8]. The most common clinical presentation is a painful groin mass; abdominal pain, nausea, vomiting, diarrhea or fever are not common. Because the narrow femoral ring usually prevents the spread of the inflammation into the abdominal cavity, patients rarely present with peritonitis [8].

Preoperative diagnosis of the de Garengeot hernia is challenging due to its rarity and lack of symptoms typically associated with acute appendicitis [3]. Thus, this hernia is often diagnosed during an emergency repair for an incarcerated hernia. Although an inguinal approach (sometimes combined with laparotomy) is the most common surgical intervention, no standard surgical procedure exists. As hernioplasty and appendectomy are required in general, accurate diagnosis of the appendix situation such as necrosis, perforation and abscess formation is very important for determining appropriate surgical strategy. In a reported case, the hernia could be diagnosed preoperatively, thereby allowing laparoscopic approach selection [4]. A meta-analysis by Linder *et al*. [1] revealed that laparoscopy was chosen in 8 of the 90 patients analyzed. In another report, four patients were treated by complete laparoscopic surgery, while TEP was adopted in only one [5]; however, unlike in our case, this case underwent an appendectomy before the mesh repair, which may increase the risk of mesh infection. Furthermore, some surgeons also adopted the transabdominal preperitoneal approach (TAPP) [6, 7]. TAPP requires a peritoneal incision for mesh placement, which may again increase the infectious risk. Infection is a major complication of de Garengeot hernia repair, with an incidence of 14–29% [4]. While recent studies report that mesh repair for an incarcerated hernia does not increase the infectious risk [9], a mesh-free repair is recommended in cases of severe inflammation due to perforation or abscess [1]. If these indications are absent, a mesh repair is possible [3, 8].

In our case, enhanced CT revealed a blind-ended structure inside the femoral canal without perforation or abscesses. We selected a one-stage laparoscopic procedure that combined TEP with appendectomy, and was expected to be less invasive and carry a lower infectious risk than other laparoscopic procedures. Incising the peritoneum around the hernia ring should be avoided to separate the mesh from the inflammatory appendix. Additionally, when planning a one-stage operation, a clean surgery should be performed before a contaminated surgery. Therefore, we decided to perform a TEP hernial repair followed by laparoscopic appendectomy. If abscess formation is unclear, a diagnostic laparoscopy can be performed before the preperitoneal approach. If the appendix is perforated, we can undertake a two-stage operation or mesh-free repair after laparoscopic appendectomy.

In conclusion, we reported a rare case of a de Garengeot hernia treated by a one-stage laparoscopic procedure. Under correct diagnosis, this TEP-first procedure could be a useful modality.

## CONFLICT OF INTEREST STATEMENT

None declared.
